# Prospects of the potential strategies to improve the efficacy of anti‐PD‐1/PD‐L1 therapy

**DOI:** 10.1002/ctm2.803

**Published:** 2022-05-19

**Authors:** Hirohito Yamaguchi, Shao‐Chun Wang, Mien‐Chie Hung

**Affiliations:** ^1^ Graduate Institute of Biomedical Sciences College of Medicine, China Medical University Taichung Taiwan; ^2^ Research Center for Cancer Biology China Medical University Taichung Taiwan

1

PD‐1 is an immune‐checkpoint regulator in T cells that transduces an inhibitory signal and inactivates T cells, while PD‐L1 is the PD‐1 ligand expressed in various cell types, including antigen‐ presenting cells (APCs) and cancer cells.^1^ The PD‐L1/PD‐1 axis plays a key role in the immune escape of cancer cells, and inhibitory monoclonal antibodies (mAbs) against PD‐1 or PD‐L1 are currently used for treatment of a wide variety of cancer types.^2^ These immune checkpoint inhibitors (ICIs) exhibit drastic therapeutic effects on a subset of patients and have revolutionized cancer therapy. However, the overall response rates are far from satisfactory due to intrinsic and acquired resistance.^3^ Therefore, in order to reduce unnecessary treatment and improve the response rates, it is an urgent need to identify the appropriate markers that can discriminate responders and non‐responders to ICIs. So far, several markers associated with the positive response to PD‐1/PD‐L1 mAbs have been proposed, including, tumour infiltrated lymphocytes (TIL), tumour mutation burden (TMB), microsatellite instability, and PD‐L1 expression in tumors.^4^ Among them, the PD‐L1 expression is used in clinic for several cancer types, and some immunohistochemistry (IHC) assays to quantify PD‐L1 expression in tumour tissues have been approved by the U.S. Food and Drug Administration (FDA).^5^ However, in multiple clinical studies, PD‐L1 expression in tumours does not correlate with clinical responses to anti‐PD‐1/PD‐L1 therapy,^4^ which is a puzzle in the field.

Recently, the puzzle seems to be resolved to some extent. PD‐L1 is highly glycosylated, and this post‐translational modification is critical for PD‐L1 protein stability and function.^6^, ^7^ PD‐L1 glycosylation is regulated by various oncogenic signalling pathways such as the epidermal growth factor receptor (EGFR) pathway, which inhibits phosphorylation of extracellular domain of PD‐L1 by GSK3β.^8^ The phosphorylation by GSK3β hinders PD‐L1 from its glycosylation, leading to its ubiquitin‐mediated proteasome degradation.^6^ In addition, another study also indicates that glycosylation of PD‐L1 interferes PD‐L1 protein detection by some traditional PD‐L1 antibodies that are designed to recognize its polypeptide antigens.^9^ Human cancer cell lines or tissues section of several cancer types treated with a glycosidase have the higher signals of PD‐L1 in IHC staining than that of the untreated one, indicating that removal of *N*‐linked glycosylation of PD‐L1 enhances binding of traditional anti‐PD‐L1 mAb to PD‐L1.^9^ Thus, it was proposed that inconsistent observations between PD‐L1 IHC staining and clinical responses may be due to the failure of accurate PD‐L1 detection in tumour tissues complicated by its glycosylation. Indeed, several studies have supported the notion that deglycosylation of tumour tissues improves predictive ability of PD‐L1 expression in tumours as a marker for anti‐PD‐1/PD‐L1 therapy.^10^, ^11^, ^12^

It is worth noting that in 2019, based on the IMpassion130 trial, the U.S. FDA granted accelerated approval to atezolizumab, a humanized anti‐PD‐L1 monoclonal antibody, in combination with nab‐paclitaxel for treatment of advanced triple negative breast cancer (TNBC) patients, whose tumours show PD‐L1 positivity by IHC tests from immune cells.^13^ However, in the following randomized Phase III clinical trial (IMpassion 131), the combination of atezolizumab and paclitaxel did not show significant clinical benefits compared to paclitaxel alone,^14^ and the atezolizumab TNBC indication was withdrawn voluntarily by Roche in 2021. In a most recent study, nine TNBC patients, who were originally excluded for atezolizumab treatment based on criteria of the IMpassion130 trial, were treated with atezolizumab and the IHC of their tumor tissues were subjected to the deglycosylation process by glycosydase in order to better measure PD‐L1 level on the tumor cells.^12^ In well consistent with the prediction, the PD‐L1 level on the surface of tumor cells after deglycosylation correlated very well to the response to atezolizumab treatment.^12^ The study cohort is relatively small; however, the correlation is highly significant. This result suggests that the failure of the IMpassion 131 Phase III trial of atezolizumab is, at least in part, due to PD‐L1 glycosylation‐mediated incorrect readout of ICH staining. Thus, a larger cohort is warrant to develop further.

In addition, through mechanism studies, many druggable targets that are involved in the resistance to ICIs were identified and the combination therapy was shown to be able to increase therapeutic efficacy and/or reverse the resistance.^8^ For instance, Tyro3 was shown to contribute to the resistance to ICI treatment through inhibition of ferroptosis that is required for T cell‐mediated cancer cell killing, providing a combination therapy of a Tyro3 inhibitor and ICIs to treat these types of resistant patients.^15^ Several markers that were shown to increase therapeutic efficacy of ICIs by combination therapy from a subset of cancer cells through different kinds of mechanisms have also provided such type of marker‐guided effective therapy (MGET).^16^, ^17^, ^18^, ^19^, ^20^, ^21^, ^22^, ^23^ Thus, appropriate markers to stratify patients for different combination therapy may pave a way to increase therapeutic efficacy for ICIs.

In conclusion, PD‐L1 detection after deglycosylation by glycosidase pre‐treatment may improve the predictive value of PD‐L1 expression as a marker to select patients for ICI treatment (Figure 1). In addition, to increase therapeutic efficacy to benefit majority of patients, MGET continues to be needed to select right patients to be treated with right combination therapy. All these are worthy of validation by further clinical trials to enhance the therapeutic efficacy of ICIs.

**FIGURE 1:**
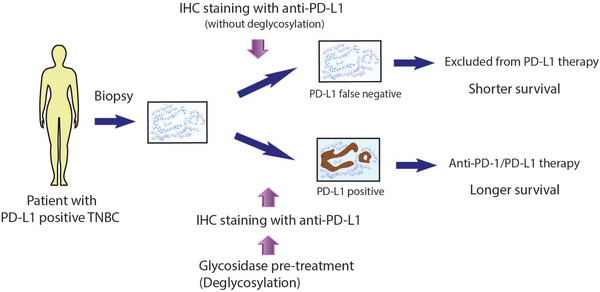
A potential strategy to improve PD‐L1 detection. TNBC tumour samples from patients are treated with glycosidase for deglycosylation, followed by immunohistochemistry (IHC) staining with anti‐PD‐L1 antibody. Compared to the conventional strategy without deglycosylation, it improves the detection of PD‐L1, thereby reducing false negative results. Thus, this strategy is expected to improve the predictive value of PD‐L1 expression as a marker to select patients for immune checkpoint inhibitors (ICI) treatment

## CONFLICT OF INTEREST

Mien‐Chie Hung holds a patent on the methodology for de‐glycosylation staining.
